# World TB Day 2016: an interview with leading experts in tuberculosis research

**DOI:** 10.1186/s12916-016-0591-9

**Published:** 2016-03-23

**Authors:** Patrick P. J. Phillips, Helen A. Fletcher, Ibrahim Abubakar, Marc C. I. Lipman, Timothy D. McHugh

**Affiliations:** MRC Clinical Trials Unit at UCL, Aviation House, 125 Kingsway, London, WC2B 6NH UK; TB Centre, London School of Hygiene & Tropical Medicine, London, UK; Centre for Infectious Disease Epidemiology, Infection and Population Health, University College London, London, WC1E 6JB UK; Medical Research Council Clinical Trials Unit, 125 Kingsway, London, WC2B 6NH UK; Royal Free London NHS Foundation Trust, London, UK; UCL Respiratory, Division of Medicine, Faculty of Medical Sciences, University College London, Royal Free Campus, London, UK; Centre for Clinical Microbiology, Division of Infection & Immunity, UCL, Royal Free Campus, Pond Street, London, NW3 2 PF UK

**Keywords:** BCG, Biomarker, Diagnosis, Transmission, Treatment, Tuberculosis, Vaccines

## Abstract

In this interview, we talk to leading tuberculosis (TB) experts from University College London and the London School of Hygiene and Tropical Medicine about the current challenges in TB research. The video of this interview is available here: https://www.youtube.com/watch?v=75Die7MQBec&feature=youtu.be. The video can also be downloaded via Additional file 1.

**Electronic supplementary material:**

The online version of this article (doi:10.1186/s12916-016-0591-9) contains supplementary material, which is available to authorized users.

## Edited transcript

### Introduction

#### Patrick Phillips (PPJP)

Tuberculosis (TB) is the leading infectious disease killer at the moment. The recent surveillance reports have showed that more people die from TB every year than from HIV or any other infectious disease. So it’s critically important that we tackle TB.

#### Helen Fletcher (HF)

Whereas people know about HIV and malaria, less people know about TB. In some ways, it is a silent pathogen. It is there in the community, it causes huge amounts of health problems, huge amounts of morbidity, and yet we have really under-invested in and neglected this disease as a global health problem.

#### Ibrahim Abubakar (IA)

If we are to contain the global burden of TB, we need new tools and diagnostics as well as new treatments and a vaccine. In order to achieve this, researchers have a key role to play.

## The re-emergence of TB

### HF

TB has always been there. We’ve never been close to eradication.

### PPJP

In Western Europe, we’ve seen something of a decline in TB over the years, but TB is still a problem in many parts of the world. There has been a particular increase in the last 10–20 years or so due to a number of factors. The emergence of the HIV epidemic – HIV knocks out a person’s immune system and makes them much more susceptible to TB – but also we have seen increasing rates of drug resistance.

### Marc Lipman (MCIL)

We also know that social cohesion in a society is incredibly important in reducing the rates of TB. This is associated with poverty and, therefore, often in parts of the world where there is civil conflict, rates of TB, as well as those for other infections, start to rise. This extremely important in terms of where TB went to – or more importantly – why it has not gone away.

## TB transmission

### IA

TB is an infectious disease acquired from person to person. It is fundamental to the control of TB that we understand transmission, because if we can stop transmission then we have a chance in ultimately eliminating TB, as is the case with the goal of the End TB Strategy. It is therefore of interest to me that we use the best technology available, whether that is whole genome sequencing, geographical mapping of cases, tools to identify clusters of cases, as well as computer programs that predict where transmission is happening, and improved statistical approaches to pinning down exactly where transmission is happening. Those are the key steps we require.

Increasingly, we are recognising that transmission does not necessarily happen in peoples’ households; that a lot of transmission, especially in high burden settings, actually happens out there in indoor-congregate community settings. Therefore, knowing exactly where these congregate settings are, and putting in interventions to stop transmission in those settings, is the key to containing TB.

## Drug resistance

### MCIL

Although we are definitely seeing an improvement in outcomes in terms of TB, we are also starting to see issues such as drug resistance. That is a direct consequence of treatment regimens that have been ineffective.

### Timothy McHugh (TMcH)

There are two aspects to why drug resistance emerges. The first is wholly about the patient and the relationship between the patient and the clinician. So it is the drugs that are given and whether the patient has been given drugs that are going to work, and whether they are the right drugs, and then whether the patient is able to take those in the way that we require them to. It might be that the drugs make them feel ill, so they don’t want to take them. Or it might be that the patient is leading a chaotic lifestyle and so isn’t able to take the drugs on a regular basis. That is what my clinician colleagues focus on – they have to manage that process.

What I’m interested in, and what my team look at, is the evolution of drug resistance – or how it emerges at a biological level for the bug itself. For me it is a question of, almost, compartments. TB is tucked away within the host, within the patient. It is a difficult site to get drugs to. Drug resistance develops when you treat an organism with not enough of the compound, so not enough drug is getting to the bug. If you’re tucked away in a compartment within the patient, where the drug isn’t going to get to, then you’re already giving a sub-optimal dose of the drug. The compounds that we use have to get over those barriers.

## Diagnostics

### TMcH

For many years we’ve used a smear test; that is, sputum put onto a slide and then used to identify *Mycobacterium tuberculosis*. This is a robust, simple tool, and has served us well for over 100 years. However, it doesn’t serve us well in the days when we need to understand drug resistance in a quick and efficient way.

### MCIL

The importance of diagnostics in the current era is that we want to be able to make a diagnosis promptly. We’ve got better and better at that. The introduction of molecular diagnostics such as GeneExpert have undoubtedly improved the sensitivity, and also the speed, of diagnosis. What’s interesting about areas such as this, is that you need both a very good test which, in general terms, can be used close to the patient – so a point-of-care test. However, you also need to re-educate your healthcare workforce, and individuals who are being tested, on the implications and value of the test.

## Treatment

### MCIL

One of the big questions about treating TB is that, because the mycobacterium is both a slow-growing organism, and also because it is relatively hardy to being killed by antimicrobials, it takes a long time to treat and effectively cure TB. For example, the standard therapy to deal with latent TB is 3 months of treatment, continuously. The standard treatment for drug-sensitive active TB is 6 months of treatment. These are drugs that have potential toxicities. That in itself is a problem because, again, if someone is feeling more unwell on the therapy, then there’s a good chance that they may stop treatment.

### PPJP

Unfortunately, we haven’t seen any improvement in the treatment of drug-sensitive TB really for 30 or 40 years. Whilst those were very effective trials and the regimen works very well in trial settings – as we’ve seen in the history of the TB epidemic, the regimen is inadequate to control the disease. So we do need treatments; we need better treatments, safer treatments, shorter treatments that are easier for patients to take.

There have been a number of trials published in the last 2 years looking at 4-month treatments. The idea being that a shorter, 4-month regimen would be easier to take for patients and, if it could be shown to be as good as the current standard of care, then that would greatly improve outcomes in practice and make it easier for TB programs to roll out treatment for a larger number of patients. Unfortunately, those three large trials show that none of the 4-month regimens being evaluated were as good as the standard of care. So we’re still looking for shorter treatments that can result in better outcomes for TB.

A lot of the work in trials at the moment is looking at how we can best identify the most promising regimens to take forward to large expensive trials. So some of the work that we’ve done, some of the research that’s being presented in the *BMC Medicine* series, is looking at better ways of doing clinical trials to identify those regimens to take forward to large Phase III trials.

## Immune correlates

### HF

At this particular time, immune correlates have emerged as an important issue because we have begun, as a TB community, to invest more in developing TB vaccines. If you look pre-clinically, there’s now quite an exciting pipeline of vaccine candidates waiting to be moved into clinical trials. What we don’t have at the moment is any real way of assessing which of those pre-clinical candidates are going to be the one that works and to go forward, because we don’t have a correlate of immune protection.

Of course, we can’t invest in efficacy testing for every one of those candidate TB vaccines. So we need tools – we need biomarkers and immune correlates – to really select the most effective vaccines and to progress the clinical development as quickly as we can. The reason we need to do this is because – if we do want to end TB by 2035 – we’re going need an effective vaccine by the year 2025.

Typically, we say it takes 10 years to develop a TB vaccine, so if we don’t start doing something about this today, we’re not going to have that vaccine candidate by 2025.

## WHO End TB Strategy

### IA

The WHO has had, together with national TB programs, great success in tackling the problem of TB. Over the last cycle of international programs that contain TB, millions – tens of millions, over 40 million – lives are estimated to have been saved. As a result of this success, and building on it in relation to the United Nations Sustainable Development Goals, the WHO has developed a new strategy which is based on the vision that we want a world that is rid of TB.

The strategy is based on three key pillars. These pillars are important, including the need to have better integrated patient-centred care, the need to have strengthening of programs and systems to support the delivery of interventions, and better research and innovation. Research and innovation is one area that those of us in universities have a key role to play and should contribute to the discovery of new tools, so that we can achieve the goals of the End TB Strategy by 2035.

## Further reading

See reference list [1–9].

## Additional file

Additional file 1: Interview with (TB) experts from University College London and the London School of Hygiene and Tropical Medicine about the current challenges in TB research. (MP4 67204 kb)
